# Measurement Invariance of the Short Home Attachment Scale: A Cross-Cultural Study

**DOI:** 10.3389/fpsyg.2022.834421

**Published:** 2022-03-11

**Authors:** Sofya Nartova-Bochaver, Sofia Reznichenko, Vasily Bardadymov, Milana Khachaturova, Victoria Yerofeyeva, Narine Khachatryan, Iryna Kryazh, Shanmukh Kamble, Zulkarnain Zulkarnain

**Affiliations:** ^1^School of Psychology, HSE University, Moscow, Russia; ^2^“Scholae Mundi Russia” Charity Foundation, Moscow, Russia; ^3^Faculty of Philosophy and Psychology, Yerevan State University, Yerevan, Armenia; ^4^Department of Applied Psychology, V.N. Karazin Kharkiv National University, Kharkiv, Ukraine; ^5^Department of Psychology, Karnatak University, Dharwad, India; ^6^Faculty of Psychology, University of Sumatera Utara, Medan, Indonesia

**Keywords:** place attachment, home attachment, questionnaire, validation, reliability, cross-cultural research

## Abstract

The home environment is a particularly significant part of life that is supposed to satisfy inhabitants’ needs, form their identity, and contribute to psychological wellbeing. The construct of home attachment is especially relevant for students as a most mobile social group. This study is devoted to the validation of *the Short Home Attachment Scale (SHAS)* in a student sample from five countries (Armenia, India, Indonesia, Russia, and Ukraine). A total of 1,349 (17–26 years; M_age_ = 19.82, SD_age_ = 2.14; 78% females) university students participated in the study and filled in the 14 items of *HAS*. In order to avoid redundant items with high error covariances damaging the model, a new scale—the *SHAS* was developed by eliminating seven items. The shortened scale has satisfactory structure validity in terms of model fit in all countries except Indonesia; internal reliability values were acceptable in all countries. Measurement invariance across countries was tested with Multi-Group Confirmatory Factor Analysis (MG CFA) and Alignment Analysis. MG CFA confirmed both configurational and metric invariance. The invariance of item factor loadings, as well as item intercepts, was also confirmed by the Alignment Analysis. The mean scores varied across cultures, with the highest in India and the lowest in Russia. The final version of *SHAS* is a valid, reliable tool that may be recommended for use in cross-cultural research. However, the *SHAS* factor structure robustness in the Indonesian population should be investigated thoroughly.

## Introduction

*East or West home is the best*

An English saying

The current paper’s aim is to develop a short cross-culturally invariant standardized tool–the *Short Home Attachment Scale (SHAS)* validated in the student sample from five cultures: Armenia, India, Indonesia, Russia, and Ukraine. Home attachment is important to study due to several long-term and ongoing changes in the lifestyles of humankind, in the first line, for intellectual youth, and students (Di Masso et al., 2019; Robinson, 2020; Rathakrishnan et al., 2021). They leave home for university and have to solve the problem of overcoming attachment to their parents’ home, establishing a new one in temporary housing–a dormitory or a rented apartment (Heidmets and Liik, 2021; Lacerda et al., 2022). Home attachment is an important factor predicting mental wellbeing, whereas homesickness is often experienced as a “mini-grief” (Stroebe et al., 2016). On the other hand, the unusual circumstances of home confinement caused by the COVID-19 pandemic have made the home a particularly important habitat for everyone, increasing the need for its arrangement as a school and a workplace. Being in lockdown was easier for those who loved their homes (Meagher and Cheadle, 2020; Ramkissoon, 2020, 2021; Counted et al., 2021). However, the latest emerging adulthood studies show that young people are returning to their parents’ homes mostly due to economic crisis (in the United States, every third young person does this) (Arnett and Schwab, 2013; Fingerman and Yahirun, 2016). So an empty nest turns into a crowded nest (Seiffge-Krenke, 2016). These features of modern life require the development of a reliable, stable tool for measuring the home attachment level.

Home attachment is a positive attitude to home manifesting in the preference for this environment over others, the desire to return there, take care of it, and keep it in the memories (Manzo and Devine-Wright, 2020; Maricchiolo et al., 2021). Since home attachment is a kind of place attachment we want to refer to the theory that describes its structure and content. There is no complete consensus on this topic. Scannell and Gifford (2010) suggested a tripartite model of place attachment including person, place, and the interaction between them. In line with this model, Hidalgo (2014) also emphasized three dimensions: person, place (social and physical levels), and psychological (affective, cognitive, and behavioral) processes. Some authors focused on place identity as a core component of place attachment (Giuliani, 2003; Williams and Vaske, 2003; Hernández et al., 2007), whereas other researchers think it may rather be a place dependence (Stokols and Shumaker, 1981; Backlund and Williams, 2003; Hernández et al., 2014). Raymond et al. (2010) and Ramkissoon et al. (2013) identified four components of place attachment: place identity, place dependence, nature bonding, and social bonding. Again, the concept of attachment links together place, nature, and people living in this place (Kyle et al., 2005; Morgan, 2010; Ramkissoon, 2021), and can be considered as a unity of emotions and activities that modulate a distance between a person and the object of attachment (Bretherton, 2013).

Home attachment is expected to differ from attachment to other objects, like a park, a city, or a tourist attraction. Being a multifunctional environment, home is responsible for inhabitants’ recreation, kinship, storage, stimulation, intimacy, and productivity (Billig, 2006; Graham et al., 2015), as well for stabilization/stimulation, support/prevention, and enhancing/ennobling (spiritualization) functions (Nartova-Bochaver et al., 2018). Home is a unity of physical, social, and existential properties of a specific place satisfying inhabitants’ needs; it means (and demonstrates) happiness, a sense of belonging, and identity. “There is an almost unanimous opinion that the prototypical place is home”; people are “domicentric” (Lewicka, 2011, p. 211). Home is a symbol of anti-chaos, stability, privacy, comfort, romance, togetherness, and security (Dmitrieva, 2014; Khachaturova and Nartova-Bochaver, 2017; Nartova-Bochaver et al., 2018; Nartova-Bochaver and Kusnetsova, 2018; Tobiasz-Lis and Wójcik, 2021), and is uniquely associated with positive feelings (McIntyre et al., 2006). Despite the agreement among scientists that the home is a most important living environment, the near-total absence of the instruments for studying home is evident. To date, there are very few standardized tools that measure the quality of the home environment or home attachment. Most of the instruments are modifications of questionnaires based on place attachment in a broad sense of this word, attenuated to a specific place, like a park or neighborhood (Williams and Vaske, 2003; Bonaiuto et al., 2006; Inglis, 2008; Boley et al., 2021) or a sense of place (Jorgensen and Stedman, 2001; Walpole et al., 2020).

The first tools to evaluate the physical features of the house were developed by Espe and Schulz (1983), Caldwell and Bradley (2003), Jansen et al. (2011), and Graham et al. (2015). However, these instruments are not standardized, long and difficult to analyze, or focused on the child environments only, and do not reflect the inhabitants’ attachment to home.

The first questionnaire measuring the level of attachment specifically to the home (the, *HAS*) was developed by Reznichenko et al. (2016). *HAS* measured a person’s emotional and functional attachment to home as an integral construct; it was a uni-dimensional scale and consisted of 14 items describing the subjective meaning of the home for its inhabitants, rated on a five-point Likert scale ranging from 1 (Strongly disagree) to 5 (Strongly agree). From that moment on, it began to be widely used in Russian-language studies.

Home attachment is a culturally sensitive phenomenon (McIntyre et al., 2006; Kavalir, 2015). Flanders (2014) distinguishes “domestic” (mainly northwestern Europe–England, Germany, and Netherlands) and “non-domestic” European cultures (mainly southern Europe–Spain, Italy, and France). Gauvain and Altman (1982) noted at least two dimensions of the home differentiating between cultures, namely identity/communality and openness/closedness. We can expect that attachment to home widely varies in conditions of the increasing diversification of family types (Georgas et al., 2006), depending on the salience of “familism” or autonomy in each culture. Therefore, for cross-cultural studies, it is important to develop a culturally invariant instrument for measuring the level of home attachment, which would reflect the stable core of this phenomenon. To our knowledge, there are no valid cross-cultural versions of home environment measures so far, except for Jones et al. (2017).

The current study presents the first five countries’ cross-cultural validation of *HAS*, in a shortened modification (*SHAS*). We expect to receive the uni-factorial structure of *SHAS* because emotions regarding a place and dependence on it are tightly interconnected (Reznichenko, 2016; Junot et al., 2018), this was proved by most previous scales, that were uni-factorial as well.

To examine *SHAS* psychometric indicators, we arranged a cross-correlational research design.

## Materials and Methods

### Participants

A total of 1,349 university students (17–26 years; Me_age_ = 19, M_age_ = 19.82, SD_age_ = 2.14; 78% females) from Armenia, India, Indonesia, Russia, and Ukraine took part in the study. After removing outliers from each subsample, the aggregate sample size was 1307: Armenia–322 participants, India–270, Indonesia–177, Russia–278, and Ukraine–260) (for the detailed information, see Supplementary Appendix 1). All students studied on university campuses away from home (M_distance_ = 439 kilometers from home) and lived mostly in dormitories or with relatives; a few (∼15%) lived in apartments rented for the duration of their studies. Participants were included in the sample if they were 17–26 years old and in an undergraduate or graduate program at the university. The exclusion criteria were respondents’ non-indigeneity or permanent rather than temporary respondents’ housing (dormitory, relative’s house, rented house) while at university.

Data were collected in 2019–2020 (see Supplementary Appendix 1). Participation was voluntary; the respondents provided some demographic information (age, sex, birthplace, and place of residence during university studies).

### Measurement Instruments

The original *HAS* items were translated into the teaching languages of the universities participated: Armenian and Indonesian, by the authors according to ISPOR requirements (Wild et al., 2005). The English version was adopted from the English questionnaire (Williams and Vaske, 2003) and modified for the home environment. As for the Armenian and Indonesian versions, these translations were made by bi-lingual psychologists who have been working for more than ten years (respectively, Armenian-Russian, and Indonesian-English specialists). After this, the back-translation was checked and approved by Dr. Reznichenko–one of the authors of the original *HAS*. All wordings were discussed with professional linguists if needed.

### Analytic Strategy

The factor structure of the questionnaire was tested step by step. The search for the optimal number of factors, as well as testing of the primary confirmatory factor analysis (CFA) model, were carried out on the data of the Russian sample (*n* = 278) since the tool was first developed in this country. The entire sample (*n* = 1,307) was used to conduct Multi-Group Confirmatory Factor Analysis (MG CFA) and Multi-group Alignment Analysis to calculate internal reliability and descriptive statistics.

We used Exploratory Graph Analysis (EGA), conducted within the glasso estimation method (graphical least absolute shrinkage and selection operator), and the Walktrap algorithm to identify the optimal number of subscales in the questionnaire.

We performed CFA with the robust maximum likelihood (MLR) rescaling-based estimator to analyze the factor structure of *HAS*. The set of commonly used goodness-of-fit indicators was used to interpret the results of both CFA and MG CFA: CFI, TLI, RMSEA, PCLOSE, and SRMR. Both CFI and TLI values exceeding 0.95 indicate a good model fit (Hu and Bentler, 1999). Value of RMSEA not greater than 0.08 and 0.06 suggests an “adequate” and “close” mode fit, respectively (Marsh et al., 2005), while SRMR values smaller than 0.08 indicate an acceptable fit (Hu and Bentler, 1999).

The internal reliability of the tool was estimated with the McDonald’s *omega* (ω) and Cronbach’s *alpha* (α; to compare the reliability across studies): both ω and α threshold values 0.70 are considered as acceptable for research purpose measurement instruments (Hair et al., 2010). The accelerated bootstrap confidence intervals for both estimates were calculated based on 1,000 bootstrap replications.

Testing of measurement invariance of the scale across countries was carried out *via* MG CFA, using the full information maximum likelihood (FIML) method. MG CFA contained three assessments of equivalence with increasing constraints: configural (no constraints), metric (constrained factor loadings), and scalar (constrained factor loadings and intercepts). Evaluation of the invariance was conducted by the assessment of changes in the fit index: ΔCFI and ΔTLI less than 0.01, ΔRMSEA less than 0.015, and ΔSRMR less than 0.03 (Chen, 2007).

It is known that scalar invariance in real research is not easy to satisfy; thus, the comparison of the factor means is often limited. In such cases, another method to test metric and scalar invariance, namely the multi-group factor analysis alignment, is more practical. The measurement alignment does not require equality restrictions on factor loadings and intercepts across groups (Asparouhov and Muthén, 2014; Fischer and Karl, 2019). Therefore, we decided that if full metric and/or scalar invariance across countries cannot be proved in the traditional MG CFA, we will choose a less demanding method. The alignment procedure was performed using a fixed approach with alignment power values specified for λ (loadings) and ν (intercepts) parameters as 0.25 and 0.25 for λ and ν tolerances set to 0.4 and 0.2, respectively.

The magnitude of the latent mean structure difference was specified using Cohen’s *d*, measuring the effect size of differences in means, where *d* greater than 0.2 is considered as a small effect, *d* = 0.5 is medium, and *d* = 0.8 or above a significant effect (Cohen, 1988).

In the current study, we used the packages psych 2.1.9 (Revelle, 2021), lavaan 0.6–9 (Rosseel, 2012), semTools 0.5–5 (Jorgensen et al., 2021), MBESS 4.8.1 (Kelley, 2021), EGAnet 1.0.0 (Golino and Epskamp, 2017), sirt 3.11–21 (Robitzsch, 2019), and ccpsyc 0.2.4 (Fischer and Karl, 2019) implemented in the R Software and Programming environment 4.1.1 (R Core Team, 2020). The calculations were performed both in Excel and R.

## Results

### Testing the Structure of the *Home Attachment Scale* in the Individual Countries

To handle missing data in the dataset (3.11% of the entire sample) the FIML method was used. Based on the calculated probability (*p* < 0.001) of the Mahalanobis distance for each observation, 42 multivariate outliers were identified and then removed from the sample (see Supplementary Appendix 1 for details). The final sample included 1,307 cases. Both the Mardia’s multivariate kurtosis and skewness tests didn’t meet the normality assumption. None of the items had a normal univariate distribution according to the Anderson-Darling test, however, the absolute values of skewness and kurtosis in each sample were between −2 and + 2, which is considered acceptable to prove normal univariate distribution (George and Mallery, 2010). Items 1, 2, 3, 4, 11, 14 showed slightly left-skewed distribution. No floor effect was detected. There was little evidence (percentage frequency of highest possible score were within 16–25%) of a ceiling effect for these items.

Exploratory Graph Analysis conducted on the Russian sample (*n* = 278) suggested the extraction of 1 cluster in the partial correlation matrix. The strongest relations were found between items 1, 3, 4, 7, 11, and 14. The results of the dimension stability analysis (based on 1,000 replica samples) confirmed that a uni-dimensional model was relatively precise: Me ± SD (CI) number of dimensions = 1 ± 0.63 (1.53); 1 factor was replicated 714 times, while 2, 3, or 4 factors only 134, 112, and 40 times, respectively. The items 8, 9, 10, and 12 had the lowest stability indices and replicated between 75 and 77% of the time in their dimension. With regard to the EGA results and original factor structure of *HAS*, a uni-dimensional solution was chosen for the CFA analysis.

The initial one-factor model performed on the Russian sample (χ^2^ = 249.42, df = 77, *p* < 0.001) showed acceptable SRMR value (0.057), but poor RMSEA (0.090 [95% CI, 0.078–0.101]; PCLOSE < 0.001) and incremental fit indices (CFI = 0.897, TLI = 0.878). The factor model was then successively reduced based on the EGA results (the most unstable items), the modification indices, and the item analysis indices (difficulty, discrimination, and item-total correlations). Items 8, 9, 10, 12, and 13 were removed first because they had (a) the lowest factor loadings (less than 0.50); (b) multiple and high error covariances between themselves and with other items, and (c) the lowest scores of item discrimination (<0.40) and item-total correlation (<0.50). These trends were fully or partially replicated in samples from all other countries. Deletion of these items led to a significant, but insufficient improvement in the model fit (RMSEA = 0.078; SRMR = 0.035; CFI = 0.949, TLI = 0.936).

**FIGURE 1:**
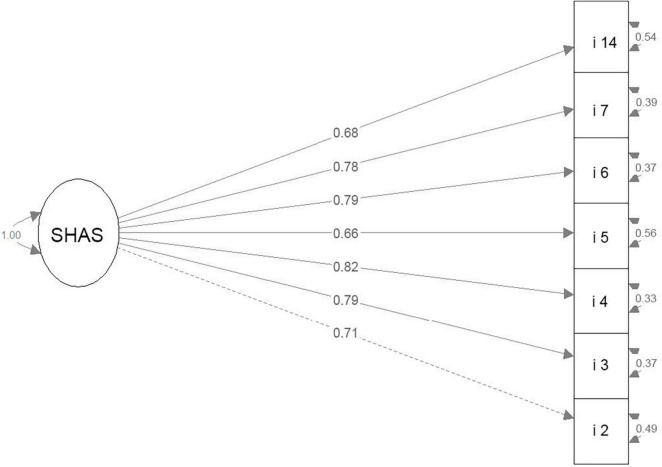
The optimal for the Russian sample CFA model tested for the *Short Home Attachment Scale*.

The modification indices showed that the sources of the residual model misspecification are high and serial error covariances between semantically close items 1–3, 1–7, 1–11, 3–7, 7–11, 3–6, and 4–14 (e.g., 1: “*I feel like my home is a part of me*”; 3:“*My home is a really special place to me*”) and that a substantial amount of misspecification can be avoided by deleting items 1 and 11. The final uni-dimensional model included seven items (2, 3, 4, 5, 6, 7, 14; see Figure 1) with the range of loadings 0.66–0.82 and fitted the Russian data perfect: χ^2^ = 22.25, df = 14, *p* = 0.074; RMSEA = 0.046 [95% CI, 0.000–0.077]; PCLOSE = 0.543, SRMR = 0.026, CFI = 0.989, TLI = 0.983.

Due to this radical shortening of the scale, it was labeled *SHAS* (see Supplementary Appendix 2). Internal consistency was satisfactory for both the CFA (Russian) sample (ω = 0.90, α = 0.90) and for other subpopulations in different countries (ω range 0.82–0.89, α range 0.82–0.89). Descriptive statistics of *SHAS* in the countries studied are shown in Table 1.

**TABLE 1:** Descriptive statistics of the *Short Home Attachment Scale* across countries.

	N	M (SD)	SE mean	Median [95%CI]	Asymmetry	Kurtosis	McDonald’s omega [95% CI]	Cronbach’s alpha [95% CI]
Armenia	322	28.12 (5.55)	0.31	29 [29–30]	−0.84	0.47	0.89 [0.87–0.92]	0.89 [0.87–0.91]
India	270	30.46 (4.32)	0.26	31 [30–32]	−1.06	0.70	0.82 [0.77–0.86]	0.82 [0.78–0.86]
Indonesia	177	27.21 (4.95)	0.37	28 [27–29]	−0.39	−0.38	0.89 [0.86–0.92]	0.89 [0.86–0.91]
Russia	278	24.29 (6.57)	0.35	25 [24–25]	−0.52	−0.02	0.90 [0.88–0.92]	0.90 [0.88–0.92]
Ukraine	260	25.76 (5.86)	0.36	26 [25–26]	−0.57	−0.27	0.84 [0.81–0.87]	0.84 [0.80–0.87]

*The median’s, McDonald’s omega’s, and Cronbach’s alpha’s confidence intervals have been estimated for each group through bootstrapping with 1,000 replicates.*

We have successfully replicated this solution both in India and Ukraine, where fit indices were excellent (RMSEA = 0.031/0.020; SRMR = 0.028/0.034; CFI = 0.993/0.996, TLI = 0.989/0.993, respectively) (see Table 2). In Armenia, we got moderate fit indices in terms of RMSEA = 0.081 and TLI = 0.942 but good fit in terms of SRMR = 0.036 and CFI = 0.961. The model could be improved by adding covariances between the errors of items 3–4 and 3–6, but these modifications led to a deterioration in the model fit of other countries, particularly Russia. Therefore, we decided not to modify the model obtained on the Russian sample and to include the Armenian data in further MG CFA because of its relatively adequate model fit. In Indonesia, the model was poor-fitted to the data (RMSEA = 0.115; SRMR = 0.044; CFI = 0.938, TLI = 0.907) and required drawing multiple, theoretically inexplicable correlations between error terms of the items 2–3, 3–6, 5–6, 3–7 2–5, 2–14, 6–7. Since the fit of the model with the data in each country is a necessary requirement for invariance, Indonesia was excluded from further analyses.

**TABLE 2:** Separate and multigroup confirmatory factor analyses of the *Short Home Attachment Scale* across countries.

Model	χ2 (df)	RMSEA [95% CI]	SRMR	CFI	TLI	Factor loadings
***Separate CFA models***						
1. Armenia	48.40 (14)***	0.081 [0.061–0.110]	0.036	0.961	0.942	0.60–0.85
2. India	15.46 (14)	0.020 [0.000–0.056]	0.034	0.996	0.993	0.52–0.72
3. Indonesia	46.88 (14)***	0.115 [0.081–0.151]	0.044	0.938	0.907	0.58–0.82
4. Russia	22.25 (14)	0.046 [0.000–0.077]	0.026	0.989	0.983	0.66–0.82
5. Ukraine	17.41 (14)	0.031 [0.000–0.069]	0.028	0.993	0.989	0.51–0.77
***Multigroup CFA models across countries***						
1. Configural invariance	102.66 (56)***	0.054 [0.039–0.069]	0.028	0.981	0.971	–
2. Metric invariance	145.52 (74)***	0.058 [0.046–0.071]	0.057	0.971	0.967	–
Δ 2-1	42.86 (18)***	0.004	0.029	−0.01	−0.004	–
3. Scalar invariance	370.73 (92)***	0.104 [0.094–0.114]	0.094	0.885	0.895	–
Δ 3-2	225.21 (18)	0.046	0.037	−0.086	−0.072	–

Data from Indonesia were excluded from the multigroup CFA. ***A chi-square difference is significant at p ≤ 0.001.

### Measurement Invariance Testing Across Countries

#### Multi-Group Confirmatory Factor Analysis

In order to examine measurement invariance of the SHAS across different cultures (except Indonesia) for further comparison of latent factor means, configural invariance, metric invariance, and scalar invariance were sequentially tested. As shown in Table 2, the configural invariance was confirmed which assumed that the overall factor structure is identical across countries. The model comparison test (configural vs. metric) suggested full metric invariance (ΔRMSEA = 0.004, ΔCFI = −0.01, ΔTLI = −0.004, ΔSRMR = 0.029), indicating that factor loadings are the same in all countries. However, scalar invariance wasn’t achieved, because all compared indicators significantly exceeded its thresholds: ΔRMSEA = 0.046, ΔCFI = −0.086, ΔTLI = −0.072, ΔSRMR = 0.037.

The effect sizes in item bias (dMACS) were calculated to check which items led to the greatest mismatch of factor models in different countries and estimate their magnitude of the misfit. Items 4 and 5 (average dMACS 0.581 and 0.689, respectively) turned out to be most problematic: it had the greatest impact both on the metric and scalar variance. The dMACS of items 3, 6, and 7, on the contrary, were the lowest (0.375, 0.262, and 0.376, respectively). The maximum dMACS values were observed in the pair of India and Armenia, and the minimum in the pair of Russia and Ukraine.

#### Multi-Group Alignment Analysis

Since we failed to establish scalar invariance of the *SHAS* using MG CFA, we used the multi-group alignment approach (Asparouhov and Muthén, 2014) to compare the latent factor means.

Table 3 displays (non)invariant countries for each item factor loading and item intercept: if a group is enclosed in parentheses, the parameter of this group is denoted as non-invariant. As can be seen, all the item factor loadings remain invariant. The intercepts of the items were more non-invariant than the loadings of the items. Armenia showed non-invariance in the intercepts of items 4 and 6, Indiaitem 7, and Russia–item 6. The percentage of non-invariance of the intercepts was 14.3% which is less than a cut-off of 25% non-invariance suggested by Asparouhov and Muthén (2014). R^2^ for loadings and intercepts were 0.99 and 1, respectively. These results indicate that essentially all non-invariance is caused by group-varying factor means and variances.

**TABLE 3:** Approximate measurement invariance (non-invariance) for groups and comparison of aligned factor means of the *Short Home Attachment Scale* across countries.

Items	Invariance (non-invariance)		Latent mean comparisons	
	for countries		across groups*	
	Loadings	Intercepts	Country	Factor mean (SD)
2	AR IN RU UK	AR IN RU UK	AR	0.681 (0.767)
3	AR IN RU UK	AR IN RU UK	IN	1.096 (0.611)
4	AR IN RU UK	(AR) IN RU UK	RU	0.000 (1.000)
5	AR IN RU UK	AR IN RU UK	UK	0.286 (0.899)
6	AR IN RU UK	(AR) IN (RU) UK		
7	AR IN RU UK	AR (IN) RU UK		
14	AR IN RU UK	AR IN RU UK		
Percentage of non-invariance item parameters	0%	14,3%		
Degree of invariance (R^2^)	0.990	0.998		

*AR, Armenia; IN, India; RU, Russia; UK, Ukraine.*

*When a group is parenthesized, the parameter of that group is indicated non-invariant.*

*differences between the latent means of the SHAS for all pairwise comparisons are significant at p ≤ 0.001 (Bonferroni-adjusted significance level for pairwise comparisons is α = 0.008).

#### Latent Mean Comparisons

Based on the multi-group alignment analysis, the latent factor means of the *SHAS* were compared. After inspecting the results, we found that the Russian sample had a smaller factor mean, so we fixed its latent mean at zero and standard deviation at one whereas the latent means and standard deviations of other groups were freely estimated (Table 3). The latent means compared by *t*-test with Bonferroni correction significantly differed across countries. Russian students had the smallest factor mean, and Indian students had the highest one [differences in means: 1.10; t(546) = 15.43; *p* > 0.001; Cohen’s *d* = 1.32]. Ukrainian students were stronger attached to their homes than Russian students [differences in means: 0.29; t(536) = 3.48; *p* = 0.001; Cohen’s *d* = 0.30], but less than Armenian, and Indian ones [differences in means: −0.40, −0.81; t(580) = −5.72 and t(528) = −12,18; all at *p* > 0.001; *d* = 0.48, 1.06, respectively]. Students from Armenia had lower SHAS scores than Indian students [differences in means: −0.42; t(590) = −7.19 at *p* > 0.001; *d* = 0.59].

## Discussion

The study aimed to examine the structural validity, measurement invariance, and reliability of *HAS* in the youth from five countries with predominantly collectivist cultures.

Consistent with the results of a previous validation of *HAS* conducted on the Russian population, the current study retained the single-factor structure of the scale. Nevertheless, the CFA results conducted on the Russian sample showed that some semantically close items of the questionnaire had high error covariances and/or low factor loadings. This led to a significant decrease in the model fit. We identified seven items that had the highest factor loadings, unique variance, and discriminative parameters and formed the most sustainable and parsimonious factor solution in the Russian sample; they were included in the final shortened version of the scale (*SHAS*). These items constitute a uni-dimensional construct of home attachment and reflect the three most frequently identified manifestations of a strong attachment to home: affect (emotions), cognition (identity), and behavior (action) (Ruiz and Hernández, 2014). This model was successfully replicated in India and Ukraine, and with relative success in Armenia where fit indices were acceptable but not perfect. In Indonesia, the model showed a poor fit to the data and required adding serial, theoretically questionable correlations between error covariances of the items’ set. Thus, *SHAS* can be used without structural modifications in Russia, Ukraine, India, and Armenia, but requires a more thorough study of the factor structure on data from the Indonesian population.

In the current study, there was evidence for both configural and metric invariance as tested by MG CFA. The invariance of item factor loadings, as well as item intercepts, was also confirmed by the alignment analysis: all the items factor loadings were the same across cultures, while the intercepts of only four items out of seven were fully invariant. However, the percentage of non-invariance (14.3%) is quite low and indicates that the structure of the questionnaire and item parameters have sufficient cross-cultural stability to compare the latent means of *SHAS* in respondents from different countries.

We could assume that home attachment is related to culture: in countries with a pronounced collectivistic orientation and a high value of family, such as India and Armenia, the highest indicators were obtained, and in countries with a moderately collectivistic orientation, such as Russia and Ukraine, lower (Hofstede Insights, 2022). This trend is consistent with several other studies showing that collectivistically orientated international students who place greater emphasis on cooperation, obligation, and respect for family values have higher levels of homesickness compared to students who endorse individualistic values because it is harder for them to tolerate reduced family presence (Hack-Polay, 2020; Poyrazli and Devonish, 2020).

## Conclusion

This paper reports the results of the structural validation of a new standardized instrument–*SHAS*, which was examined in five countries (Armenia, India, Indonesia, Russia, and Ukraine). The results show that the aim of our research has been achieved, and now, researchers have a new concise and convenient method of studying the personal attitudes to home environment.

Nevertheless, the current study is not free of some limitations; the most important of them might be overcome through examining the content, discriminant, and convergent validity; a more detailed study of the factor structure and modification indices of the questionnaire on Indonesian data; further exploration of the age dynamics in home attachment, widening the number of participants from individualistic cultures; extending the sample by recruiting different social groups, for instance, work migrants, refugees, homeless people; and implementing the research results in the abroad social context and practice. This is on the agenda for future studies.

Despite these limitations of the current study, the new method can be recommended for cross-cultural research, especially for homelessness, homesickness, adaptation to a new (temporary or permanent) place of residence, and also used in applied research, such as motivation for mobility and tourism.

## Data Availability Statement

The datasets analyzed in this study are available online in the OSF repository (10.17605/OSF.IO/9GM4W).

## Ethics Statement

The studies involving human participants were reviewed and approved by the Commission for the Ethical Evaluation of Empirical Research Projects of the Department of Psychology at HSE University. The patients/participants provided their written informed consent to participate in this study.

## Author Contributions

SN-B developed the main idea of the manuscript, collected data, organized the database, wrote the first draft of the manuscript, contributed to the manuscript revision, read and approved the submitted version. SR contributed to the study’s conception and design, performed the statistical analysis, contributed to the manuscript revision, read and approved the submitted version. VB, MK, VY, NK, IK, SK, and ZZ collected data, organized the database, provided feedback, read and approved the submitted version. All authors approved the submitted version of the manuscript.

## Conflict of Interest

The authors declare that the research was conducted in the absence of any commercial or financial relationships that could be construed as a potential conflict of interest.

## Publisher’s Note

All claims expressed in this article are solely those of the authors and do not necessarily represent those of their affiliated organizations, or those of the publisher, the editors and the reviewers. Any product that may be evaluated in this article, or claim that may be made by its manufacturer, is not guaranteed or endorsed by the publisher.
