# Supplementary material for: Measurement Invariance of the Short Home Attachment Scale: A Cross-Cultural Study
Source: Front Psychol. 2022 Mar 11;13:834421. doi: 10.3389/fpsyg.2022.834421 (PMC8961976; doi:10.3389/fpsyg.2022.834421)
Supplement: Supplementary file 1 [file Data_Sheet_1.pdf]

Appendix 1

Description of the samples

Country	N of participants after removal of outliers (N of excluded participants)	Age: M ± SD; range (years)	Sex: female (%)	Place of residence: a dormitory / relative's house / rented house (%)	Way of data collection and language
Armenia	322 (11)	19.92 ± 1.36; 18–26	78.21	90.45 / 4.78 / 4.77	online, via 1ka.si service, in Armenian
India	270 (16)	22.36 ± 1.38; 19–26	68.23	96.39 / 0.36 / 3.25	pencil-and-paper, in person, in English
Indonesia	177 (5)	18.41 ± 0.62; 17–20	78.57	1.10 / 67.58 / 31.32	pencil-and-paper, in person, in Indonesian
Russia	278 (6)	19.07 ± 0.86; 17–23	77.19	45.96 / 32.63 / 21.41	online, via 1ka.si service, in Russian
Ukraine	260 (4)	18.29 ± 0.69; 17–21	81.06	39.02 / 46.97 / 14.01	online, via Google-form service, in Russian
p-value		0.001	> 0.001	> 0.001	
Effect size		Partial eta squared = 0.51	φ = 0.12	Cramer’s V = 0.48	

Note. *Age comparison was calculated via ANOVA. The comparisons of sex and place of residence were calculated via* χ^2 *statistics.*
