# Supplementary material for: Measurement Invariance of the Short Home Attachment Scale: A Cross-Cultural Study
Source: Front Psychol. 2022 Mar 11;13:834421. doi: 10.3389/fpsyg.2022.834421 (PMC8961976; doi:10.3389/fpsyg.2022.834421)
Supplement: Supplementary file 2 [file Data_Sheet_2.pdf]

**Appendix 2**

The *Short Home Attachment Scale*: Extended English, and Corresponding Reduced English and Russian Versions

**Инструкция:**

Опросник измеряет отношение людей к своему дому. Пожалуйста, оцените степень вашего согласия с каждым утверждением, поставив ✓ в поле с наиболее подходящим вариантом. Пожалуйста, ответьте на каждое утверждение, основываясь на собственных ощущениях и опыте.

**Instruction:**

The questionnaire measures the attitude of people to their home. Please rate your level of agreement with each statement by placing a ✓ in the box with the most appropriate option. Please answer each statement based on your own feelings and experience.

| Item code | The *Home Attachment Scale*<br>English Wording | The *Short Home Attachment Scale*<br>English Wording | The *Short Home Attachment Scale*<br>Russian Wording |
|---|---|---|---|
| 1 | I feel like my home is a part of me | - | - |
| 2 | My home is the best place for what I like to do | My home is the best place for what I like to do | Мой дом — лучшее место заниматься тем, что мне нравится |
| 3 | My home is a really special place to me | My home is a really special place to me | Мой дом — это особенное место для меня |
| 4 | No other place can compare to my home | No other place can compare to my home | Ни одно другое место не может сравниться с моим домом |
| 5 | I identify strongly with my home | I identify strongly with my home | Мой дом и я очень похожи друг на друга |
| 6 | I get more satisfaction from being in my home than from visiting any other place | I get more satisfaction from being in my home than from visiting any other place | Я получаю большее удовлетворение, находясь в собственном доме, нежели в других местах |
| 7 | I am very attached to my home | I am very attached to my home | Я очень привязан(а) к своему дому |

| | | | |
|---|---|---|---|
| 8 | Doing what I do in my home is more important to me than doing it in any other place | - | - |
| 9 | Those who visit my home can learn a lot about me | - | - |
| 10 | While doing my favorite things at home, I enjoy them no less than if I did it somewhere else. | - | - |
| 11 | My home means a lot to me | - | - |
| 12 | I wouldn't substitute any other place for doing the types of things I do in my home | - | - |
| 13 | I identify with the lifestyle and values of the people who live in my home | - | - |
| 14 | I am ready to invest my heart and soul into my home | I am ready to invest my heart and soul into my home | Я готов(а) вкладывать силы и душу в дом, где я живу |

*Note.* Responses were made on a seven-point scale: from *1* (Совершенно не согласен(а); Strongly disagree) to *5* (Полностью согласен(на); Strongly agree).
